# The impact of COVID-19 on HIV treatment of adolescents in sub-Saharan Africa: A scoping review

**DOI:** 10.4102/hsag.v28i0.2226

**Published:** 2023-09-27

**Authors:** Yolanda R. Mayman, Brian van Wyk

**Affiliations:** 1 Department of Psychology, Faculty of Community and Health Sciences, University of the Western Cape, Cape Town, South Africa; 2 School of Public Health, Faculty of Community and Health Sciences, University of the Western Cape, Cape Town, South Africa

**Keywords:** COVID-19, adolescents, HIV, antiretroviral therapy, treatment outcomes adherence, social support, economic impact

## Abstract

**Background:**

Adolescents living with HIV are a key population who are susceptible to poor health. The global coronavirus disease 2019 pandemic and widespread national COVID-19 restrictions has disrupted health service delivery and HIV support services, affecting treatment adherence among adolescents with HIV.

**Aim:**

This study aimed to review the available literature on the impact of the COVID-19 pandemic on the HIV treatment of adolescents in sub-Saharan Africa.

**Method:**

Seven online databases were searched for articles published between 2020 and 2022 that focused on the impact of COVID-19 on adolescents living with HIV on antiretroviral therapy. A data charting extraction form and the Preferred Reporting Items for Systematic Reviews and Meta-Analyses Protocol (PRISMA) flowchart were used for screening and reporting the articles in this review. A narrative synthesis was conducted.

**Results:**

Five overarching themes emerged from the articles in this review, which highlighted the mental, social, and economic impacts of the COVID-19 pandemic, as well as the impact of the reallocation of healthcare services and challenges to accessing HIV care services on the antiretroviral therapy (ART) adherence of adolescents living with HIV.

**Conclusion:**

The global COVID-19 pandemic affected adolescents living with HIV in sub-Saharan Africa in many ways, but very little research has been done to describe the various ways in which the physical and mental well-being of adolescents were impacted.

**Contribution:**

The findings of this review can be used to further inform policies and interventions aimed at the care and well-being of adolescents on antiretroviral therapy within sub-Saharan Africa.

## Introduction

In December 2019, an illness of unknown origin, later named the SARS-CoV-2 or coronavirus, was first detected in Wuhan, China (Dzinamarira et al. 2021). On 27 February 2020, the first COVID-19 case was reported in sub-Saharan Africa (Lone & Ahmad 2020). In Africa, over 9.3 million COVID-19-related deaths have been reported and confirmed as of 26 September 2022 (World Health Organization [WHO] 2022). The impact of COVID-19 has been recorded to have exacerbated poverty, widespread hunger, and domestic violence (Gittings et al. 2021). Severe and additional economic and social inequalities continue to negatively impact both the psychosocial and treatment outcomes of vulnerable groups such as adolescents living with HIV (Armbruster et al. 2020; Dyer et al. 2021).

The COVID-19 pandemic and subsequent lockdown measures have impeded access to clinical care, delaying positive individuals’ access to HIV treatment and care (Dorward et al. 2021; Nyoni & Okumu 2020). An already over-burdened public health sector in sub-Saharan Africa means that public health services are inadequately resourced and experience a shortage of healthcare workers (Dorward et al. 2021; Nyoni & Okumu 2020). Severe economic and social inequalities and disruptions to HIV services have been described as impacting vulnerable and marginalised populations such as adolescents living with HIV (Armbruster et al. 2020; Enane et al. 2022).

The short- and long-term impact of COVID-19 crosses biological, psychological, and social domains (Chenneville et al. 2020). Biologically, individuals living with HIV are at an increased risk of experiencing complications related to COVID-19 and the further progression of the HIV disease (Chenneville et al. 2020). Psychologically it is argued that individuals with HIV may experience a stronger stress response in comparison to the rest of the population because of the increased risk of contracting COVID-19 (Chenneville et al. 2020; Nanni et al. 2015; Xiong et al. 2020). Individuals living with HIV may also experience a more severe social impact, as they may be more reluctant to physically interact with others, leading to a disintegration of social bonds and a loss of support gained from HIV support groups.

The majority (89%) of the 1.6 million adolescents living with HIV globally reside in sub-Saharan Africa (Okumu, Nyoni & Byansi 2021). The HIV epidemic disproportionately affects groups from communities heavily impacted by social determinants of health, such as poverty, unemployment, racial discrimination, and stigma (Armbruster et al. 2020). Adolescents and young adults living with HIV aged 10–24 years are more vulnerable to experiencing poor health outcomes in every phase of the HIV care continuum: testing, diagnosis, medication adherence, and viral suppression (Armbruster et al. 2020; Dyer et al. 2021; Okumu et al. 2021). This group also experiences a higher rate of loss to follow-up, virological failure, and mortality in comparison to adults living with HIV and receiving HIV care (Dyer et al. 2021). Not only do these inequalities negatively impact the health of adolescents and youth living with HIV but also increase their risk of and susceptibility to HIV transmission within their groups and communities (Armbruster et al. 2020).

## Rationale for the review

Adolescents living with HIV face structural challenges such as the unavailability of safe centres for healthcare and support, which impacts their antiretroviral therapy (ART) adherence (Brown et al. 2000; Goga et al. 2020). Moreover, strategies implemented during the COVID-19 pandemic to prevent the spreading of the coronavirus resulted in poorer mental health among adolescents (Goga et al. 2020). The review may provide valuable insights into the experiences of adolescents living with HIV during the COVID-19 pandemic, while informing further research, which could enable health systems to better respond to future health emergencies and societal disruptions.

## Significance of the review

Vrazo et al. (2020) mentioned that there is a lack of data on the impact of the COVID-19 pandemic on healthcare systems, particularly the delivery of HIV services. The significance of this review is therefore highlighted as it provides a synthesis of the current literature available on the impacts of COVID-19 on adolescents living with HIV as a vulnerable and key population in combating the HIV pandemic in sub-Saharan Africa. In addition, sub-Saharan Africa also has the biggest proportion of adolescents living with HIV globally. The aim of this review is thus to add to the gap, which exists and to inform further research and the development of treatment interventions to further promote and sustain treatment adherence.

## Methods

### Scoping review

This scoping review used the five steps as described by Arksey and O’Malley (2005) and Mak and Thomas (2022). The five steps include: (1) identifying the review question, (2) identifying relevant studies, (3) selecting studies, (4) charting data, and (5) summarising and reporting findings.

### Identifying the review question

The review question for this scoping review is: *What is the impact of the COVID-19 pandemic on the HIV treatment of adolescents in sub-Saharan Africa?*

### Identifying relevant studies

#### Inclusion criteria

The studies were included, if:

- The study sample included adolescents aged between 10 and 19 years. The sample is described as youth, children, young people, teens, adolescents, young adults, or a group that could include these.
- Focus of the study included the impact of COVID-19 on ART.
- Topics related to adolescents living with HIV and AIDS were discussed.
- Topics related to the experiences of adolescents during the COVID-19 pandemic were discussed.
- Studies conducted in sub-Saharan African countries.
- Original research articles published between 2020 and 2022.

#### Search strategy for identification of studies

A comprehensive database search of literature reporting on the impact of COVID-19 on adolescent adherence to ART was conducted in seven databases: PubMed, SCOPUS, CINAHL, ERIC, PsycINFO, CABI Direct, and African Index Medicus. Full-text articles were obtained using search strings containing keywords using the ‘AND’ and ‘OR’ Boolean operators where appropriate. The search terms included ‘adolescent’, ‘adherence’, ‘antiretroviral therapy’, ‘COVID-19’, and ‘HIV’ and ‘ART’.

#### Process of selecting sources of evidence

**FIGURE 1:**
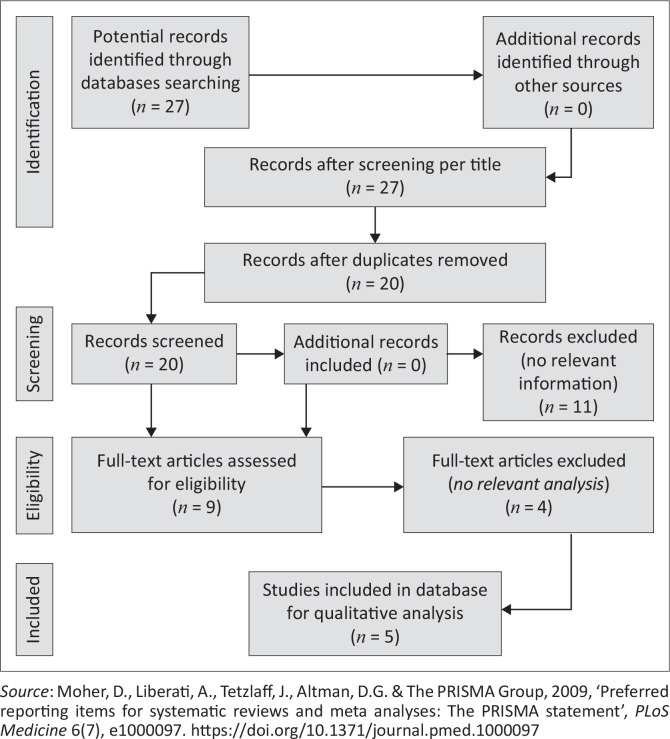
Preferred Reporting Items for Systematic Reviews and Meta-Analyses flow diagram for studies selection.

Studies were selected based on the inclusion and exclusion criteria formulated for the scoping review. The inclusion criteria were based on the Population, Concept, and Context (PCC) framework, where P = adolescents living with HIV; C = impact of the COVID-19 pandemic, and C = sub-Saharan Africa. The Preferred Reporting Items for Systematic Reviews and Meta-Analyses Extension for Scoping Reviews (PRISMA-ScR) flowchart by Tricco et al. (2021) was used for screening and reporting the articles in this review as seen in Figure 1. A total of 27 articles were identified and titled screened through database searching. Once the duplicates were 20 articles were abstract screened, and of these 11 articles were excluded because they failed to meet the inclusion criteria. Nine articles went through full-text screening and a final total of five articles were included in this review. A two-stage screening process of the articles took place independently by two reviewers and there were no disagreements.

### Charting data: Summarising and reporting findings

#### Data charting

A data extraction form for the charting of data from the included articles was used. Both reviewers developed the form and independently checked the data items included to ensure that the items aligned with the aim and review question of the review. Data items included the author(s), the country in which the study was conducted, the study design or article type, participant characteristics and/or setting, sample size, study objectives, ethics, and the main findings of the included articles. Table 1 details the data extraction information of the included articles.

**TABLE 1:** Characteristics of included studies.

Study 1D.	First author Year	Country	Study design or article type	Participant characteristics and/or setting	Sample size	Study objectives	Main findings
1.	Dyer et al. (2021)	Kenya	Survey	ALHIV ages 10–24 years receiving HIV care at nine health facilities in Western Kenya.	486	To assess the psychosocial effects of COVID-19 on ALHIV To assess the feasibility of conducting behavioural surveys by phone.	Findings showed adolescents experienced difficulties refilling their ART medication, subsequently missing their ARV doses. Additionally, ALHIV in the study also reported economic challenges such as unemployment because of the lockdown restrictions and food shortages, which impacted their medication regimens. The findings of this study also demonstrate that conducting surveys by phone is feasible with ALHIV in rural Kenya. Offering remote peer support or mental healthcare, continuing to offer differentiated care services, and considering financial support will support the health and well-being of ALHIV.
2.	Enane et al. (2021)	Kenya	Survey	Study participants included adolescents between the ages of 10–19; enrolled in care at one of the study sites, and attended at ≥1 visit in the 18 months before study enrolment	334	To assess a range of effects of the pandemic on ALHIV in western Kenya To assess whether effects were greater for ALHIV with recent histories of being lost to the programme (LTP).	The COVID-19 pandemic has had devastating socioeconomic effects on Kenyan ALHIV and their households. ALHIV with recent care disengagement may be especially vulnerable. Sustained ART access and adherence potentially signal resilience and strengths of ALHIV and their care programmes. Most ALHIV in this study, including those in the LTP cohort, had access to ART, which may reflect either recent care re-engagement and/or the efforts of the care programme and the study team to ensure ART access.
3.	Vrazo et al. (2020)	Kenya, Burundi, Uganda, Eswatini, Malawi, Mozambique, Tanzania, Zimbabwe, Cameroon, DRC, Zambia, Nigeria, Lesotho, South Africa	Commentary	Pregnant and breastfeeding women, infants, and C/ALHIV	0	To propose whole-family, tailored programme adaptations along the HIV clinical continuum to account for the needs of pregnant and breastfeeding women, infants, and C/ALHIV	Protecting pregnant and breastfeeding women, infants, children, and adolescents from acquiring SARS-CoV-2 while sustaining essential HIV services is an immense global health challenge. Tailored, family-friendly programme adaptations for case-finding, ART delivery, and viral load monitoring for these populations have the potential to limit SARS-CoV-2 transmission while ensuring the continuity of life-saving HIV case identification and treatment efforts.
4.	Van Staden et al. (2022)	South Africa	Commentary	ALHIV in South Africa	0	To reflect on the effects of COVID-19 on the South African healthcare services, and social and educational support structures. To assess its impact on ART adherence and retention in HIV care among ALHIV.	Adolescent HIV care in South Africa is often overlooked; however, ART adherence among ALHIV in South Africa is particularly susceptible to the consequences of a world transformed by COVID-19. The current structures in place to support HIV testing, ART initiation, and adherence have been reshaped by disruptions to health structures, new barriers to access to health services, and the limited available education and psychosocial support systems. Reflecting on these limitations can drive considerations for minimising these barriers and retaining ALHIV in HIV care.
5.	Chenneville et al. (2022)	Kenya	Descriptive phenological	YLWH in Kenya	40	To examine the impact of COVID-19 on YLWHIV in Kenya	Various individual, community, healthcare and government factors were identified as impacting YLWH. Results show that YLWH experienced difficulties accessing HIV care and treatment. Furthermore, inadequate support, the economic impact of COVID-19, and disruptions to healthcare impacted the mental and physical well-being of YLWH.

Note: Please see the full reference list of the article, Mayman, Y.R. & Van Wyk, B., 2023, ‘The impact of COVID-19 on HIV treatment of adolescents in sub-Saharan Africa: A scoping review’, *Health SA Gesondheid* 28(0), a2226. https://doi.org/10.4102/hsag.v28i0.2226, for more information.

ALHIV, adolescents living with HIV; C/ALHIV, children or adolescent living with HIV; YLWH, youth living with HIV; ART, antiretroviral therapy.

#### Data synthesis

The findings of this review were described using narrative synthesis. A narrative synthesis is the description and summary of the various findings of the synthesis. According to Lisy and Porritt (2016), the use of textual narrative synthesis allows for the investigation of resemblances and variances between the studies, the exploration of potential relationships within the data, and a summary of acquired information that can be used to inform future research and policy. The steps outlined in a narrative synthesis include developing a preliminary synthesis of the findings of included studies, exploring the relationships within and between the studies, and assessing the robustness of the synthesis (Ryan 2013).

### Ethical considerations

Ethical clearance was obtained from Biomedical Science Research Ethics Committee (reference number: BM22/7/4). This forms part of a larger project. Participant consent was not obtained because this study had no participants.

## Results

### Characteristics of included studies

The included studies align with the aims and objectives of this review and were written by experts in this field. The quality appraisal of articles was not performed in this review because of the divergent article types and different study designs of the included studies. According to the JBI Manual for Scoping Reviews (Peters et al. 2021), the quality assessment of articles is not required for scoping reviews as the aim of a scoping review is to identify and map the nature and extent of research evidence. Two articles included in this review were surveys (Dyer et al. 2021; Enane et al. 2020), two studies were commentaries (Van Staden et al. 2022; Vrazo et al. 2020) and one was a qualitative study (Chenneville et al. 2022). The geography of the studies includes African countries such as Kenya, Burundi, Uganda, Eswatini, Malawi, Mozambique, Tanzania, Zimbabwe, Cameroon, DRC, Zambia, Nigeria, Lesotho and South Africa.

The sample sizes varied between the three research studies. Dyer et al. (2021) had a sample of 486 participants, with 152 being adolescents living with HIV (ALHIV) between the ages of 10 years and 14 years, 188 adolescents living with HIV between the ages of 15 years and 19 years, and 146 young adults between 20 and 24 years. Enane et al. (2020) had a sample of 334 adolescents living with HIV between the ages of 10 years and 19 years. Chenneville et al. (2022) had a sample of 40 participants, with 15 being youth between the ages of 18 years and 24 years, living with HIV, 13 youth between the ages of 18 years and 24 years, affected by HIV, and 12 healthcare providers living with HIV.

### Summary of themes

Five overarching themes emerged from the articles in this review, these are:

- the mental impact of COVID-19 on adolescents living with HIV;
- the economic impact of COVID-19 on adolescents living with HIV;
- the social impact of COVID-19 on adolescents living with HIV;
- reallocation of healthcare resources and challenges to accessing HIV care services during the COVID-19; and
- ART adherence of adolescents living with HIV (see Table 1).

### The mental health impact of COVID-19 on adolescents living with HIV

The restrictions imposed in response to the COVID-19 pandemic variously impacted the mental health state of adolescents living with HIV. Van Staden et al. (2022) found that adolescents face an increased risk of mental health challenges such as depression during the pandemic. In the study conducted by Enane et al. (2021), the scores of the adolescents showed that 5.6% of the participants scored 3 or higher on the Patient Health Questionnaire-2 (PHQ-2) scale – indicating possible depression. In addition, 5.2% of adolescents living with HIV in this study also scored 3 or higher on the General Anxiety Disorder-2 (GAD-2) scale, indicating potential anxiety. Dyer et al. (2021) found that 1% of the adolescents reported moderate-to-severe depression symptoms on the PHQ-2 scale. The study by Chenneville et al. (2022) found that the multiplicative social effects of the pandemic exacerbated other illnesses or diseases and mental health issues, which are comorbid with HIV.

### The economic impact of COVID-19 on adolescents living with HIV

Chenneville et al. (2022) found that financial issues caused by the COVID-19 pandemic included unemployment, decreased income, and increased medication, and nutrition costs. In the study conducted in Kenya by Enane et al. (2021:5) it is reported that ‘prior to the pandemic, 35 (10.5%) of 334 adolescents were working or earning income, primarily in the informal sector and mostly in public-facing jobs’. However, since the start of the COVID-19 pandemic, 26 (74.3%) of 35 adolescents lost their jobs and income, which diminished their means to buy food (Enane et al. 2021). Moreover, over 35% of adolescents in this study relied on someone else within their household who also lost their job and income during the pandemic. Van Staden et al. (2022:3) assert that a ‘40% increase in unemployment among South African adults between February and April 2020 led to approximately 3 million South Africans falling below the poverty line’. Unemployment and subsequent financial stress negatively affect the ability of caregivers to provide support for adolescents living with HIV. The study by Dyer et al. (2021:70) in Kenya found that the most common economic effects of the pandemic reported by participants were ‘losing employment, not being able to access their job, or business being slow’. This decrease in income impacted caregivers’ ability to provide food which increased food scarcity and hunger.

### The social impact of COVID-19 on adolescents living with HIV

The social impacts of the pandemic were highlighted in three of the included articles. Enane et al. (2021:20) found that ‘being out of school, missing socialization with peers, lacking money for food or basic needs, and a lack of work for themselves or their families’ were the major life challenges adolescents experienced during the pandemic. Additionally, the commentary by Van Staden et al. (2022:3) found that ‘evidence-based support structures were less accessible to adolescents during the most restricted lockdown periods’. Similarly, findings by studies conducted in Kenya by Dyer et al. (2021) and Chenneville et al. (2022) found that adolescents and youth living with HIV and/or AIDS experienced social isolation, fear, and relationship issues because of curfew restrictions.

### Healthcare resource reallocation and challenges to accessing HIV care services during the COVID-19 pandemic

Van Staden et al. (2022) highlighted that from 2005–2008 to 2015–2016 there had been a substantial increase in adolescents living with HIV (ALHIV) aged between 15 years and 19 years receiving ART treatment. However, the relocation of healthcare workers to assist in the management of COVID-19 screening and testing resulted in less HIV testing in children and adolescents during the initial lockdown period. As a result, adolescents were unable to gain access to HIV care and treatment during the pandemic. According to Van Staden et al. (2022), it is necessary to understand the barriers to accessing ART and challenges experienced by adolescents. This can help to ascertain the broader impact of the COVID-19 pandemic and ensure the ‘continuation of HIV healthcare services and adherence support for adolescents living with HIV’ (Van Staden et al. 2022:3). Enane et al. (2021) reported that 17% of adolescents in the study conducted in Western Kenya were concerned about running out of ART medication during the pandemic. Similarly, in the study by Dyer et al. (2021) 17% of adolescents were no longer able to go to their local clinic to get medical care and unable to get medication refills.

According to Chenneville et al. (2022:5), the difficulties accessing treatment as expressed by youth living with HIV included ‘barriers to accessing medical services, having to avoid hospital due to fears of contracting COVID-19, transportation difficulties and inadequate medication supplies’. Additionally, Van Staden et al. (2022:3) expressed that the COVID-19 pandemic has hindered the support that caregivers provide adolescents during clinic visits as ‘older or at-risk caregivers avoided health facilities’. The COVID-19 pandemic has also affected the quality and quantity of HIV care and services. Van Staden et al. (2022) state that because of a reduction in socialisation, personal protective equipment, and limited interaction time between clients and providers, adolescents living with HIV are no longer able to engage in crucial services. The disruption to adherence to counselling may also increase HIV transmission, unsafe sex, and re-infection.

### Antiretroviral therapy adherence of adolescents living with HIV

Enane et al. (2021) reported that adolescents’ ART adherence was negatively affected as a result of forgetting, not taking their antiretroviral drugs (ARVs) because of there being no food, not wanting to take ARV around others, and making changes to their schedules because of the pandemic or their living situations. According to Vrazo et al. (2020), service delivery adaptations such as multi-month ARV dispensing and community-based ART services are potentially impactful interventions for individuals who are initiating or continuing ART treatment. Community-based ART approaches are impactful in terms of retention rates among vulnerable groups, including ALHIV. However, the lack of community and support services rendered children and adolescents living with HIV who were unstable on ART and have failing regimens at increased morbidity and mortality. This places children and adolescents living with HIV in an increasingly vulnerable position and they should thus be prioritised by clinical support and virtual case management.

Van Staden et al. (2022) point out that interventions such as multi-month dispensing have been introduced to lessen ART interruption. However, they may not be effective as ALHIV require constant support and monitoring to ensure adherence and retention. There is thus a need for community-level support groups, promotion of HIV medication adherence and the ongoing HIV epidemic within the context of the COVID-19 pandemic, gainful employment, and the distribution of personal protective equipment (PPE) and providing education, awareness, and funding geared towards HIV stigma reduction (Chenneville et al. 2022).

## Discussion

Mental challenges are often associated with low ART adherence, poor retention in HIV care and overall poor HIV treatment outcomes (Haas et al. 2023). A systematic review of studies in sub-Saharan Africa found that individuals with HIV who present significant depression symptoms had a 55% less chance of achieving ART adherence compared with those without significant depression symptoms (Nakimuli-Mpungu et al. 2011). According to Laurenzi et al. (2020), mental health challenges such as depression, anxiety, hopelessness and fear for the future are common among adolescents living with HIV. Another systematic review on studies in sub-Saharan Africa on the health-related needs among adolescents living with HIV on ART reported that mental health issues such as depression and anxiety among adolescents with HIV were attributed to HIV-related stigma from society, which can be classified as an adherence barrier (Chem et al. 2022). Additional research conducted in South Africa found that adolescents with HIV experienced emotional challenges linked to depression, anxiety, stress and sadness as a result of their experiences of the lockdowns and uncertain futures (Gittings et al. 2021; Moreno et al. 2020). This review found that the COVID-19 pandemic negatively impacted the mental health of adolescents living with HIV in South Africa, and possibly affected their ability to adhere to ART in this period.

Economic barriers are often interrelated with other biopsychosocial barriers and pose a barrier to optimal adherence among adolescents with HIV (Hlophe et al. 2023; Kansiime et al. 2021). A systematic review of studies in South Africa found that food insecurity is common in most low-socioeconomic settings in South Africa and poses a barrier to adolescents taking their ART medication as prescribed (Muchena & Kalenga 2021). Ahmed et al. (2023) found that COVID-19 lockdowns, service restrictions and adjustments increased food insecurity and poverty in many communities. Desmond, Sherr and Cluver (2021), Gittings et al. (2021) and Niles et al. (2020) observed that not having food led to many adolescents living with HIV not adhering to their HIV medication during the COVID-19 pandemic.

A study by Cluver et al. (2021) found evidence that social relationships within healthcare settings and communities are key factors and predictors of quality of care and healthcare outcomes. Findings from this review show that social and travel restrictions resulted in adolescents living with HIV being forced to relocate, thereby disrupting and limiting their social interactions with others. Research by Tomlinson et al. (2021) posits that social factors such as social isolation, the loss of loved ones, the disruption of school programmes, food insecurity, and access to healthcare services all directly and indirectly impact the overall adherence of adolescents living with HIV. This finding is echoed in a study conducted in Malawi, which found that adolescents identified a lack of parental, peer or sibling support as a barrier to adherence.

The HIV-related health services were significantly reduced because of the reallocation of staff and resources to managing COVID-19 outbreaks (Chenneville et al. 2021). A shortage in health workers meant that fewer healthcare workers were available to monitor adolescents living with HIV on ART and conduct follow-ups. The lack of HIV services also meant that adolescents living with HIV lost a support structure, which increased feelings of hopelessness and helplessness, and in turn decreased their motivation to stay adherent to HIV treatment during the pandemic (Muchena & Kalenga 2021). In addition, the national responses to curb the spread of COVID-19 disrupted the support services that adolescents received, further highlighting the structural challenges experienced by adolescents living with HIV (Van Wyk & Mayman 2022).

Kim et al. (2014) posit that as poor ART adherence increases, the risk of treatment efficacy decreases, which may lead to disease progression. Findings from this review show that factors such as limited contact with healthcare staff, limited access to healthcare sites for ART medication refills, a lack of social and medical support and structural changes as a result of the pandemic negatively impacted the treatment adherence of adolescents living with HIV. It is therefore necessary that research be undertaken to further explore the impact of the COVID-19 global pandemic on the ART adherence of ALHIV.

## Review limitations

This review is not without limitations as there is a low number of articles included in this review as the focus of this study was on the impact of COVID-19 on adolescents living with HIV within sub-Saharan African countries. As a result of the inclusion criteria of this study and the amount of research conducted, it is possible that relevant studies were missed. The inclusion of more articles may have further strengthened the findings of this study. However, it is clear that the COVID-19 pandemic has impacted and continues to impact the well-being and treatment outcomes of ALHIV.

## Conclusion

The period of adolescence poses very unique challenges to ART adherence, which renders adolescents as a vulnerable group (Kim et al. 2014; Van Wyk & Davids 2019). Findings from this review highlight the need for further in-depth exploration on the impact of the COVID-19 pandemic on the HIV treatment experiences and challenges of adolescents in sub-Saharan Africa. The disruption in HIV clinical and support services during the pandemic calls for a restoration of these services as well as a recovery from the losses caused by this interruption. Further research should also be directed towards learning from the health systems responses to the COVID-19 pandemic, and how to navigate future outbreaks and pandemics. This can ensure that the impact of future pandemics on vulnerable populations such as ALHIV is minimised.
